# Underdiagnosed and Misunderstood: Clinical Challenges and Educational Needs of Healthcare Professionals in Identifying Autism Spectrum Disorder in Women

**DOI:** 10.3390/bs15081073

**Published:** 2025-08-07

**Authors:** Beata Gellert, Janusz Ostrowski, Jarosław Pinkas, Urszula Religioni

**Affiliations:** School of Public Health, Centre of Postgraduate Medical Education of Warsaw, 01-826 Warsaw, Poland

**Keywords:** autism spectrum disorder, ASD, women, gender differences, diagnostic challenges, social camouflaging, psychiatric comorbidities

## Abstract

Autism Spectrum Disorder (ASD) remains significantly underdiagnosed in women, resulting in a persistent gender gap with important clinical, functional, and psychosocial implications. This narrative review explores the multifactorial barriers contributing to diagnostic disparities, including the male-oriented structure of current diagnostic criteria, the prevalence of co-occurring psychiatric conditions, and the phenomenon of social camouflaging shaped by culturally reinforced gender norms. These factors frequently lead to delayed identification, clinical misinterpretation, and suboptimal care. The review synthesizes evidence from clinical, psychological, and sociocultural research to demonstrate how the under-recognition of ASD in women impacts mental health outcomes, access to education, occupational stability, and overall quality of life. Special emphasis is placed on the consequences of missed or late diagnoses for healthcare delivery and the educational needs of clinicians involved in ASD assessment and care. This article concludes with actionable, evidence-based recommendations for enhancing diagnostic sensitivity, developing gender-responsive screening strategies, and integrating training on female autism presentation into medical and allied health education. Addressing these challenges is essential to reducing diagnostic inequities and ensuring timely, accurate, and person-centered care for autistic women throughout their lifespan.

## 1. Introduction

Autism Spectrum Disorder (ASD) is a heterogeneous group of neurodevelopmental conditions characterized by persistent difficulties in social communication and interactions, as well as restricted and repetitive patterns of behavior, interests, or activities, as defined by both the DSM-5-TR and ICD-11 classification systems (American Psychiatric Association, 2022). While the clinical definition is uniform, the manifestation of symptoms and their recognition in real-world contexts often vary significantly between individuals—particularly across genders.

Current research consistently reports that ASD is diagnosed substantially more often in males than in females, with male-to-female ratios commonly cited around 3:1, although some studies suggest even wider disparities (Posserud et al., 2021). However, a growing body of evidence challenges the assumption that these numbers reflect true prevalence differences. Instead, the discrepancy is increasingly attributed to limitations in diagnostic frameworks and a longstanding neglect of gender-specific presentations of autism (Milner et al., 2023; Hull et al., 2020).

Standard diagnostic tools, including the Autism Diagnostic Observation Schedule (ADOS-2) and the Autism Diagnostic Interview—Revised (ADI-R), were developed and validated primarily using male samples. As a result, they are less sensitive to the more nuanced behavioral patterns often observed in autistic females (Tsuji et al., 2022). The so-called “female autism phenotype” is frequently marked by subtle social difficulties, advanced masking or camouflaging strategies, and internalized distress. Furthermore, autistic women show elevated rates of psychiatric comorbidities such as anxiety, depression, and eating disorders—conditions that may obscure core autism traits and lead to misdiagnosis or delayed recognition (Tsuji et al., 2022; Navarro-Pardo et al., 2021; Stroth et al., 2022).

Consequently, many autistic women remain unidentified until adolescence or adulthood, often after years of ineffective treatments targeting co-occurring symptoms rather than the underlying condition. This delay not only hampers access to appropriate support, but also increases the risk of long-term psychosocial difficulties, including educational underachievement, social isolation, occupational challenges, and a reduced quality of life (Navarro-Pardo et al., 2021; Stroth et al., 2022).

The purpose of this article is to critically examine the current state of knowledge regarding the diagnostic and functional challenges experienced by women with ASD. Through a narrative review of the classical and contemporary literature, including perspectives from autistic individuals themselves, we aim to identify the key factors contributing to underdiagnosis and misdiagnosis. This article concludes with evidence-based recommendations for improving diagnostic equity, fostering gender-sensitive clinical practices, and informing future directions in research, education, and support systems.

## 2. Diagnostic Frameworks and Clinical Limitations

Women on the autism spectrum are disproportionately underdiagnosed or misdiagnosed due to multiple overlapping factors, including the male-centered structure of the diagnostic criteria, high rates of psychiatric comorbidities that obscure autistic traits, and gender-based social expectations. One of the most significant challenges is the historical development of diagnostic tools and frameworks based predominantly on male populations, resulting in a clinical model that fails to adequately capture how autism manifests in girls and women (Edwards et al., 2024; Gu et al., 2023). Standardized instruments such as the Autism Diagnostic Observation Schedule and the Autism Diagnostic Interview—Revised frequently lack sensitivity to the subtle presentation of ASD in females, particularly in those with average or above-average intellectual functioning who may engage in masking behaviors or mimic neurotypical social patterns. However, studies have shown that these tools, originally developed and normed primarily on male samples, may fail to detect autism in women who present with more internalized symptoms or use compensatory strategies (Ratto, 2021). This limitation stems from the fact that diagnostic frameworks and instruments were historically constructed based on relatively homogenous, male-dominated samples. As a result, individuals who do not conform to these prototypes, such as autistic women, may remain undiagnosed or misdiagnosed. While valuable in clinical practice, their limitations underscore the need for complementary assessments that better capture gendered expressions of autism.

In this context, it is also worth emphasizing the commonness of the phenomenon social camouflaging—a prominent feature of the female autism phenotype, in which individuals consciously or unconsciously imitate neurotypical behaviors to navigate social situations (Hull et al., 2021). While this strategy may result in a superficial appearance of social competence, it often conceals significant internal distress and reduces the likelihood of receiving an ASD diagnosis. Diagnostic assessments that rely heavily on structured observation or rigid behavioral checklists are especially prone to overlooking these masked difficulties. Moreover, existing diagnostic frameworks such as the DSM-5-TR and ICD-11 often fail to reflect the lived experiences of autistic women, particularly when co-occurring psychiatric conditions further obscure core symptoms. In addition to camouflaging, autistic females may also present with sensory processing differences (e.g., heightened sensitivity to noise or touch) and restricted interests that align with socially acceptable topics, such as animals, video games, or literature. These interests, although socially appropriate on the surface, may still cause significant functional impairment when intense and inflexible, yet they are frequently overlooked during diagnostic evaluations (Tsuji et al., 2022; Edwards et al., 2024).

Emerging instruments, such as the Girls Questionnaire for Autism Spectrum Conditions (GQ-ASC), attempt to address this gap by including items that reflect gender-specific experiences, such as identity confusion, social mimicry, and anxiety related to gender roles (Napolitano et al., 2022). However, it is important to point out that the GQ-ASC is not yet widely implemented and remains outside standard diagnostic practice. Its strengths lie in its targeted focus on the female autism phenotype, offering items that capture subtle presentations often missed by generic tools. Limitations include a lack of normative data and validation across diverse populations, which hinders its broader clinical adoption. Additionally, the effective use of the GQ-ASC requires specific training for clinicians to interpret its results accurately within a comprehensive diagnostic framework. While other gender-sensitive tools are in development, the GQ-ASC remains a primary focus due to its advanced stage of design and empirical support.

Improving the diagnostic accuracy for women with ASD may benefit from broadening the scope of evaluation beyond traditional tools. Complementary approaches, such as qualitative interviews, informant reports, and ecological observations have been suggested in the literature as potentially valuable for capturing subtler or more context-dependent traits. Additionally, the use of flexible, gender-sensitive criteria may help clinicians better recognize diverse presentations of autism. Supporting this process through appropriate training, particularly in identifying gendered manifestations and using validated instruments across varied populations, remains an important area for development.

## 3. Psychiatric Comorbidities and Sociocultural Masking

Another major diagnostic barrier is the high prevalence of psychiatric comorbidities among autistic women, which frequently obscure or delay the recognition of ASD. These include mood and anxiety disorders, eating disorders, and obsessive–compulsive disorder (OCD), each of which may dominate the clinical picture and divert attention from autism as the underlying condition.

Mood and anxiety disorders are among the most frequently reported co-occurring conditions in individuals with autism spectrum disorder. However, recent meta-analytical findings suggest that the prevalence of depressive disorders in autistic populations is lower than previously estimated. In a synthesis of 100 studies, the pooled prevalence of depressive disorders was approximately 11%, while anxiety disorders were present in about 20% (Lai et al., 2019; McCrossin, 2022). These estimates, though lower than earlier clinical assumptions, remain higher than in the general population and vary across study types, age, gender, and settings.

Prevalence rates from clinical samples are notably higher than those from population-based data, suggesting that the psychiatric burden may be greatest among those seeking care. For autistic women, this issue is compounded by late or missed diagnoses. Many report long-standing mental health difficulties beginning in childhood, often misattributed to other conditions. Chronic internal distress, misunderstood social challenges, and repeated mislabeling frequently lead to cascades of inaccurate diagnoses—including depression, anxiety, eating disorders, bipolar disorder, borderline personality disorder, and PTSD.

The evidence suggests that many psychiatric symptoms reflect cumulative trauma linked to undiagnosed neurodivergence (Lai et al., 2019; McCrossin, 2022). Without understanding the underlying autistic profile, women often receive fragmented care that treats individual symptoms rather than the root condition. This impedes self-understanding and access to appropriate support.

These findings underscore the need to recognize autism as a key upstream factor in women’s mental health. ASD is not a mental illness, but without appropriate identification, women often endure years of misdiagnosis and ineffective interventions. Early recognition can be transformative (Lai et al., 2019).

Another key comorbidity is eating disorders, especially anorexia and bulimia (Schröder et al., 2025). Restrictive eating behaviors are over-represented in autistic females. For some, anorexia functions as a coping mechanism for sensory overload or social unpredictability. Autism traits—cognitive rigidity, obsessive interests, and sensory sensitivities—overlap with eating disorder symptoms, complicating the diagnosis. Clinicians may misinterpret these features as anorexia-specific, missing the underlying autism.

Similarly, OCD is common. Compulsions may create a sense of predictability, but differentiating them from autism-related repetitive behaviors is difficult (Jacob et al., 2009). The overlap often obscures both conditions, leading to inadequate care. Overall, psychiatric comorbidities pose a major diagnostic challenge, where secondary diagnoses overshadow autism and hinder proper intervention.

Sociocultural factors also contribute to underdiagnosis (Wierenga et al., 2023). Expectations around femininity like empathy or social compliance can mask autistic traits. Girls described as “shy” or “creative” are less likely to be flagged than boys (Brickhill et al., 2023; Salari et al., 2022). Those with superficial peer relationships may go unnoticed, despite internal distress. Conformity to gender norms fosters camouflaging strategies, such as mimicking expressions, social scripts, or tone (Hull et al., 2021; Green et al., 2019). Though helpful in the short term, camouflaging is mentally exhausting and linked to burnout.

Systemically, diagnosis is limited by clinicians’ lack of training in female ASD presentations. Most manuals and tools are based on male-biased research (Calderoni, 2023). Many women do not meet classic criteria, yet face serious functional difficulties. Additionally, diagnostic systems focus on early childhood, leaving adult women undiagnosed, often after years of misdirected treatment. Formal diagnostic pathways for adult women remain scarce and access to multidisciplinary teams is limited.

## 4. Female Presentation of ASD

Difficulties in social functioning are a core characteristic of autism spectrum disorder and are central to both clinical definitions and lived experiences of autistic individuals. However, as outlined in previous sections, the manifestation of these difficulties in women often deviates from the stereotypical, male-associated profile upon which most diagnostic tools are based. As a result, the subtle, internalized, or atypical ways in which autistic traits present in females frequently go unrecognized by clinicians, educators, and even the individuals themselves. This diagnostic gap, compounded by psychiatric comorbidities and sociocultural expectations, contributes to a chronic underdiagnosis and misdiagnosis of autism in women.

Building on this foundation, the following section explores how social functioning challenges uniquely manifest in autistic women, extending beyond the clinical setting into daily life. It also highlights the psychological costs of camouflaging behaviors, the tension between internal experiences and external expectations, and the often-overlooked implications for identity, autonomy, and mental health. In addition to social and communicative features, the discussion also considers gendered expressions of restricted and repetitive behaviors—core diagnostic elements of ASD that may present differently in women. By deepening the understanding of gender-specific expressions of autism in social, educational, and occupational domains, this discussion aims to contribute to a more inclusive and accurate conceptualization of autism spectrum disorder—one that reflects the full range of autistic experiences across gender lines.

As we underlined in the previous paragraphs, difficulties in social functioning are one of the most common characteristics of autism spectrum disorder. However, in autistic women, these difficulties often manifest in atypical and less overt ways, making them less likely to be recognized by professionals, caregivers, or peers. Unlike the classic male ASD profile, which frequently involves obvious social withdrawal, autistic girls and women often engage in persistent, though frequently ineffective, attempts at social connection. This behavior is described in the literature as “social clinginess” or “relational mimicry” (Kopp & Gillberg, 1992).

A key mechanism underlying social functioning in autistic women is social camouflaging, which includes both conscious and unconscious strategies for concealing autistic traits. These may involve mimicking neurotypical behavior, memorizing conversation scripts, imitating facial expressions and intonation, and suppressing emotional or sensory needs (Hull et al., 2021). Although camouflaging can lead to a superficially appropriate social presentation, it often lacks emotional authenticity and requires immense cognitive and emotional effort. Over time, this effort contributes to psychological distress, including anxiety, identity confusion, and exhaustion.

Importantly, camouflaging is not the only trait that can mask autism in women. Other characteristics, such as restricted and repetitive behaviors or intense interests, may also be present, but manifest differently than in men. Autistic women are more likely to engage in restricted interests that are perceived as socially normative, such as interests in animals, literature, pop culture, or fictional characters, but pursue them with a depth, rigidity, and emotional intensity that may disrupt daily life. These behaviors may not raise clinical concern because they appear age-appropriate or gender-conforming, yet they serve similar regulatory or cognitive functions as stereotyped behaviors observed in autistic men. Similarly, sensory sensitivities (a hallmark of ASD) are often internalized or misattributed to anxiety or mood disorders in women, further complicating recognition. As with camouflaging, these gendered expressions can complicate diagnosis and contribute to internalized stress when the intensity of such traits is misunderstood or dismissed.

Studies indicate that many autistic women experience social relationships as confusing, emotionally exhausting, and difficult to navigate (Navarro-Pardo et al., 2021; Tint & Weiss, 2018). Despite being perceived as sociable or well-adjusted, they often report profound challenges in understanding social norms, reading nonverbal cues, and maintaining a stable sense of self within interpersonal contexts. Many autistic women simultaneously desire connection and experience it as overwhelming or unsatisfying. This ambivalence may lead to social anxiety, dependency on asymmetric relationships, or the avoidance of intimacy altogether. Prolonged social camouflaging is also associated with autistic burnout—a chronic state of physical, emotional, and cognitive fatigue arising from continuous masking and overadaptation. Burnout is linked to increased depressive and anxiety symptoms, diminished executive functioning, and emotional dysregulation.

Blurred interpersonal boundaries are another hallmark of social functioning in autistic women. Some may adopt others’ behaviors, beliefs, or identities, leading to identity diffusion and heightened vulnerability to exploitation or manipulation (Tint & Weiss, 2018). Relationships may be characterized by submissiveness, dependency, or difficulty recognizing harmful dynamics. These social patterns are often under-recognized in clinical settings, but have important implications for mental health and autonomy.

Autistic women navigate social environments shaped by strong and often contradictory gender norms. Cultural expectations of femininity—including emotional expressiveness, verbal fluency, esthetic attention, and relational empathy—stand in stark contrast to the core social–communicative challenges of ASD. In contrast to autistic men, who may be socially excused from conforming to normative emotional expression, autistic women face persistent pressure to embody neurotypical ideals of womanhood.

This pressure often results in two opposing responses. Some women internalize societal norms and intensify camouflaging efforts, leading to the chronic suppression of their authentic identity. Others reject conventional roles, experiencing early detachment from gender norms and developing alternative identity frameworks (Edwards et al., 2024; Wood-Downie et al., 2021). This divergence can be accompanied by confusion, social marginalization, or psychological distress.

Body-related discomfort is another under-recognized aspect of the autistic female experience. The avoidance of gendered clothing, bras, makeup, or high heels is frequently misinterpreted as neglect or noncompliance, when it often reflects sensory hypersensitivity or incongruence with self-image. This disconnect between gendered expectations and personal experience contributes to a higher prevalence of gender identity variance and sexual orientation diversity among autistic individuals—particularly women. Research shows that autistic females are more likely to identify as non-binary, LGBTQ+, or experience gender dysphoria (Rea et al., 2024). These identity dimensions may compound feelings of alienation and increase vulnerability to stigma or misunderstanding within both professional care systems and social networks.

What is also important is that educational and occupational challenges in autistic women remain underexplored in the literature, despite their significant impact on quality of life (Morrison et al., 2025). Many autistic women possess high cognitive and verbal abilities, yet struggle to fully realize their potential due to social challenges, sensory overload, and a lack of individualized support.

In educational settings, autistic girls are often described as “quiet,” “mature,” or “well-behaved,” and are thus overlooked by teachers and clinicians. They tend to perform well academically, masking underlying emotional strain and social difficulties. Studies show elevated levels of perfectionism, chronic anxiety, difficulty with group work, and the avoidance of socially flexible activities. During adolescence, social demands increase, and many autistic girls experience heightened exclusion, loneliness, or even school withdrawal (Dean et al., 2023).

In the labor market, autistic women face substantial structural and social barriers (Hayward et al., 2018). Difficulties begin during the recruitment phase—nonverbal communication, ambiguity in job expectations, and self-presentation requirements often disadvantage autistic candidates. Soft skills such as adaptability, assertiveness, and teamwork are especially challenging. Many autistic women report experiencing cognitive overload, difficulty managing change, and burnout in fast-paced or high-pressure environments. In response, some pursue remote work, freelance roles, or highly structured tasks that better accommodate their sensory and cognitive needs. However, these adaptations may come at the cost of social integration or professional advancement.

Importantly, many women do not associate their difficulties in school or work with autism. Instead, they are often treated for depression, anxiety, or chronic fatigue without the recognition of underlying neurodivergence. This misattribution leads to treatment misalignment and prolongs suffering, while delaying access to meaningful support.

## 5. Future Directions

To address the diagnostic inequities faced by autistic women, future efforts must proceed on several inter-related fronts.

First, there is a need for the systematic refinement and validation of diagnostic tools that adequately capture the female autism phenotype. Instruments such as the Girls Questionnaire for Autism Spectrum Conditions (Brown et al., 2020) represent promising advances, but must be integrated into standard practice and supported by normative data and clinician training. Diagnostic protocols should include both a structured assessment and open-ended clinical inquiry, incorporating informant reports and contextual observations across developmental stages.

Second, professional education must be expanded to include comprehensive training on gender-specific manifestations of ASD. Curricula for physicians, psychologists, educators, and allied health professionals should emphasize the subtleties of camouflaging, the masking of autistic traits through compensatory behaviors, and the clinical implications of misattributed psychiatric comorbidities. Specific training content should include the recognition of gendered presentations, differential diagnosis, trauma-informed care, and a culturally responsive assessment. Training programs should be delivered through accessible and varied formats, such as interactive workshops, online modules, and interdisciplinary case-based seminars. To ensure effectiveness, evaluation metrics, such as pre- and post-training assessments, clinical decision-making simulations, and follow-up evaluations of diagnostic accuracy should be integrated. Interdisciplinary collaboration is critical to ensure a nuanced, person-centered assessment that reflects current evidence and practice.

Third, support structures should be designed to reflect the lived experiences of autistic women, particularly those diagnosed in adolescence or adulthood. Gender-informed therapeutic models, peer mentoring programs, and psychoeducation that affirms both neurodiversity and gender diversity can contribute to improved psychological outcomes and adaptive functioning.

Fourth, future research must prioritize inclusivity, representation, and intersectionality. Diagnostic disparities cannot be fully addressed without acknowledging how gender interacts with other identity dimensions such as one’s race, ethnicity, socioeconomic status, and cultural background. Women from marginalized communities may face additional barriers to diagnosis due to clinician bias, stigma, or unequal access to healthcare. Therefore, prospective longitudinal studies should explore the diagnostic experiences of autistic women from diverse backgrounds and investigate how intersecting social determinants of health impact recognition and care. Importantly, autistic women should be meaningfully involved in the design, conduct, and dissemination of the research as co-investigators, advisors, and authors.

Addressing the under-recognition of autistic women is not merely a clinical challenge, but an ethical imperative. Ensuring diagnostic equity and responsive care requires not only improved tools and training, but also a fundamental shift toward valuing the diverse ways in which autism is experienced and expressed across gender and sociocultural contexts.

## 6. Conclusions

This narrative review has synthesized current knowledge on the underdiagnosis and misdiagnosis of ASD in women, highlighting how gendered differences in symptom presentation, clinical recognition, and diagnostic pathways contribute to persistent disparities in care.

The review identified three core areas of concern. First, diagnostic frameworks and tools remain insufficiently sensitive to the female autism phenotype. Standardized assessments, developed and validated largely in male populations, often fail to detect camouflaging behaviors, internalized distress, sensory sensitivities, and restricted interests that manifest differently in women. Second, psychiatric comorbidities and sociocultural expectations frequently mask autistic traits in women. High rates of anxiety, depression, eating disorders, and the misattribution of symptoms to other psychiatric conditions delay or prevent accurate diagnosis. Third, the review revealed how functional challenges in autistic women, including difficulties in social relationships, educational settings, the workplace, and in identity formation are compounded by diagnostic delays and a lack of gender-sensitive support.

Across these domains, the review emphasizes that autistic women are at increased risk of being overlooked not due to an absence of symptoms, but because their symptoms often do not conform to the male-oriented models embedded in diagnostic systems. This has significant consequences for their mental health, autonomy, and access to appropriate care.

To bridge this diagnostic gap, this review presents several actionable recommendations. These include refining and validating gender-responsive diagnostic instruments, expanding clinician training on gendered manifestations of ASD, and ensuring inclusive, intersectional approaches to assessment and support. The involvement of autistic women as co-creators in research and education is also highlighted as a necessary step toward more equitable care.

In conclusion, recognizing and responding to the unique ways autism presents in women is not merely a clinical refinement, but also it is a public health and ethical imperative. A shift toward gender-informed practices is essential to ensure timely, accurate, and person-centered care for autistic women across the lifespan.

## Data Availability

All data are available from the corresponding author.

## References

[B1-behavsci-15-01073] American Psychiatric Association (2022). Diagnostic and statistical manual of mental disorders.

[B2-behavsci-15-01073] Brickhill R., Atherton G., Piovesan A., Cross L. (2023). Autism, thy name is man: Exploring implicit and explicit gender bias in autism perceptions. PLoS ONE.

[B3-behavsci-15-01073] Brown C. M., Attwood T., Garnett M., Stokes M. A. (2020). Am I autistic? Utility of the girls questionnaire for autism spectrum condition as an autism assessment in adult women. Autism in Adulthood.

[B4-behavsci-15-01073] Calderoni S. (2023). Sex/gender differences in children with autism spectrum disorder: A brief overview on epidemiology, symptom profile, and neuroanatomy. Journal of Neuroscience Research.

[B5-behavsci-15-01073] Dean M., Chang Y. C., Shih W., Orlich F., Kasari C. (2023). Social engagement and loneliness in school-age autistic girls and boys. Women’s Health.

[B6-behavsci-15-01073] Edwards H., Wright S., Sargeant C., Cortese S., Wood-Downie H. (2024). Research review: A systematic review and meta-analysis of sex differences in narrow constructs of restricted and repetitive behaviours and interests in autistic children, adolescents, and adults. Journal of Child Psychology and Psychiatry.

[B7-behavsci-15-01073] Green R. M., Travers A. M., Howe Y., McDougle C. J. (2019). Women and autism spectrum disorder: Diagnosis and implications for treatment of adolescents and adults. Current Psychiatry Reports.

[B8-behavsci-15-01073] Gu Z., Dawson G., Engelhard M. (2023). Sex differences in the age of childhood autism diagnosis and the impact of co-occurring conditions. Autism Research.

[B9-behavsci-15-01073] Hayward S. M., McVilly K. R., Stokes M. A. (2018). Challenges for females with high-functioning autism in the workplace: A systematic review. Disability and Rehabilitation.

[B10-behavsci-15-01073] Hull L., Cook J., Hull L., Crane L., Mandy W. (2021). Camouflaging in autism: A systematic review. Clinical Psychology Review.

[B11-behavsci-15-01073] Hull L., Lai M. C., Baron-Cohen S., Allison C., Smith P., Petrides K. V., Mandy W. (2020). Gender differences in self-reported camouflaging in autistic and non-autistic adults. Autism.

[B12-behavsci-15-01073] Jacob S., Landeros-Weisenberger A., Leckman J. F. (2009). Autism spectrum and obsessive-compulsive disorders: OC behaviors, phenotypes and genetics. Autism Research.

[B13-behavsci-15-01073] Kopp S., Gillberg C. (1992). Girls with social deficits and learning problems: Autism, atypical Asperger syndrome or a variant of these conditions. European Child & Adolescent Psychiatry.

[B14-behavsci-15-01073] Lai M. C., Kassee C., Besney R., Bonato S., Hull L., Mandy W., Szatmari P., Ameis S. H. (2019). Prevalence of co-occurring mental health diagnoses in the autism population: A systematic review and meta-analysis. The Lancet Psychiatry.

[B15-behavsci-15-01073] McCrossin R. (2022). Finding the true number of females with autistic spectrum disorder by estimating the biases in initial recognition and clinical diagnosis. Children.

[B16-behavsci-15-01073] Milner V., Colvert E., Mandy W., Happé F. (2023). A comparison of self-report and discrepancy measures of camouflaging: Exploring sex differences in diagnosed autistic versus high autistic trait young adults. Autism Research.

[B17-behavsci-15-01073] Morrison C., Cashin A., Foley K. R. (2025). Daily living skill support for autistic people through a neurodiversity-affirming practice lens. Australian Occupational Therapy Journal.

[B18-behavsci-15-01073] Napolitano A., Schiavi S., La Rosa P., Rossi-Espagnet M. C., Petrillo S., Bottino F., Tagliente E., Longo D., Lupi E., Casula L., Valeri G., Piemonte F., Trezza V., Vicari S. (2022). Sex differences in autism spectrum disorder: Diagnostic, neurobiological, and behavioral features. Frontiers in Psychiatry.

[B19-behavsci-15-01073] Navarro-Pardo E., López-Ramón F., Alonso-Esteban Y., Alcantud-Marín F. (2021). Diagnostic tools for autism spectrum disorders by gender: Analysis of current status and future lines. Children.

[B20-behavsci-15-01073] Posserud M. B., Skretting Solberg B., Engeland A., Haavik J., Klungsøyr K. (2021). Male to female ratios in autism spectrum disorders by age, intellectual disability and attention-deficit/hyperactivity disorder. Acta Psychiatrica Scandinavica.

[B21-behavsci-15-01073] Ratto A. B. (2021). Commentary: What’s so special about girls on the autism spectrum?—A commentary on Kaat et al. (2020). Journal of Child Psychology and Psychiatry.

[B22-behavsci-15-01073] Rea H. M., Øien R. A., Webb S. J., Bansal S., Strang J. F., Nordahl-Hansen A. (2024). Gender diversity, gender dysphoria/incongruence, and the intersection with autism spectrum disorders: An updated scoping review. Journal of Autism and Developmental Disorders.

[B23-behavsci-15-01073] Salari N., Rasoulpoor S., Shohaimi S., Jafarpour S., Abdoli N., Khaledi-Paveh B., Mohammadi M. (2022). The global prevalence of autism spectrum disorder: A comprehensive systematic review and meta-analysis. Italian Journal of Pediatrics.

[B24-behavsci-15-01073] Schröder S., van Elburg A., Spek A., Danner U. (2025). Eating behaviors of autistic women with an eating disorder. Nutrients.

[B25-behavsci-15-01073] Stroth S., Tauscher J., Wolff N., Küpper C., Poustka L., Roepke S., Roessner V., Heider D., Kamp-Becker I. (2022). Phenotypic differences between female and male individuals with suspicion of autism spectrum disorder. Molecular Autism.

[B26-behavsci-15-01073] Tint A., Weiss J. A. (2018). A qualitative study of the service experiences of women with autism spectrum disorder. Autism.

[B27-behavsci-15-01073] Tsuji Y., Matsumoto S., Saito A., Imaizumi S., Yamazaki Y., Kobayashi T., Fujiwara Y., Omori M., Sugawara M. (2022). Mediating role of sensory differences in the relationship between autistic traits and internalizing problems. BMC Psychology.

[B28-behavsci-15-01073] Wierenga L. M., Ruigrok A., Aksnes E. R., Barth C., Beck D., Burke S., Crestol A., van Drunen L., Ferrara M., Galea L. A. M., Goddings A. L., Hausmann M., Homanen I., Klinge I., de Lange A.-M., Geelhoed-Ouwerkerk L., van der Miesen A., Proppert R., Rieble C., Bos M. G. N. (2023). Recommendations for a better understanding of sex and gender in the neuroscience of mental health. Biological Psychiatry: Global Open Science.

[B29-behavsci-15-01073] Wood-Downie H., Wong B., Kovshoff H., Mandy W., Hull L., Hadwin J. A. (2021). Sex/gender differences in camouflaging in children and adolescents with autism. Journal of Autism and Developmental Disorders.

